# Dynamic predictions from longitudinal CD4 count measures and time to death of HIV/AIDS patients using a Bayesian joint model

**DOI:** 10.1016/j.sciaf.2022.e01519

**Published:** 2023-01-02

**Authors:** Feysal Kemal Muhammed, Denekew Bitew Belay, Anne M Presanis, Aboma Temesgen Sebu

**Affiliations:** a College of Natural Science, Hawasa University, P.O.Box:05, Hawasa, Ethiopia; b College of Science, Bahir Dar University, Bahir Dar, Ethiopia; c MRC Biostatistics Unit, University of Cambridge, UK; d Department of Statistics, Haramaya University, Dire Dawa, Ethiopia

**Keywords:** Joint model, Dynamic predictions, Longitudinal, Time-to-event, Bayesian model averaging

## Abstract

A Bayesian joint modeling approach to dynamic prediction of HIV progression and mortality allows individualized predictions to be made for HIV patients, based on monitoring of their CD4 counts. This study aims to provide predictions of patient-specific trajectories of HIV disease progression and survival. Longitudinal data on 254 HIV/AIDS patients who received ART between 2009 and 2014, and who had at least one CD4 count observed, were employed in a Bayesian joint model of disease progression. Different forms of association structure that relate the longitudinal CD4 biomarker and time to death were assessed; and predictions were averaged over the different models using Bayesian model averaging. The individual follow-up times ranged from 1 to 120 months, with a median of 22 months and IQR 7–39 months. The estimates of the association structure parameters from two of the three models considered indicated that the HIV mortality hazard at any time point is associated with the rate of change in the underlying value of the CD4 count. Model averaging the dynamic predictions resulted in only one of the hypothesized association structures having non-zero weight in almost all time points for each individual, with the exception of twelve patients, for whom other association structures were preferred at a few time points. The predictions were found to be different when we averaged them over models than when we derived them from the highest posterior weight model alone. The model with highest posterior weight for almost all time points for each individual gave an estimate of the association parameter of –0.02 implying that for a unit increase in the CD4 count, the hazard of HIV mortality decreases by a factor (hazard ratio) of 0.98. Functional status and alcohol intake are important contributing factors that affect the mean square root of CD4 measurements.

## Introduction

Globally new HIV infections have started to decrease steadily over the last 10 years. International efforts to improve HIV prevention and treatment services also minimize HIV transmission. As with AIDS-related mortality, the decrease in new HIV infections between 2010 and 2018 was the highest in Eastern and Southern Africa (28% decline) [1,2]. Global decreases in AIDS-related deaths have largely been seen in sub-Saharan Africa, especially in Eastern and Southern Africa, which consists 53% of the world’s population live with HIV. AIDS-related mortality has decreased by 42% between 2010 and 2017 in Eastern and Southern Africa, indicating the rapid rate of increase in treatment in the region [3,4].

Ethiopia has been severely affected by the HIV epidemic for the last three decades and there are an increasing number of people living with HIV/AIDS and taking antiretroviral therapy (ART). Although overall estimates of HIV prevalence in the general population remain low, with the 2016 Ethiopian Demographic and Health Survey reporting a prevalence of adult HIV of 0.9%, there are major differences by region (4.8% in Gambella, 3.4% in Addis Ababa, and 0.4% in SNNPR), and by type of region (2.9% urban versus 0.4% rural). According to the latest UNAIDS Spectrum-derived estimates, 613,803 people were living with HIV (PLHIV) in Ethiopia by the end of 2017, of whom 443,213 (72%) were on treatment [5].

There has been an increasing interest in personalized medical research in recent years, including in the HIV field. Monitoring of a biomarker of disease progression can allow doctors to customize treatment decisions to the characteristics of the patient, to improve medical care and survival [6]. For example, monitoring a HIV patient’s CD4 counts allows a clinician to decide when to start and adjust anti-retroviral treatment [7]. However, analysing a longitudinal biomarker measuring disease progression, such as CD4 count, and a survival outcome separately can lead to biased estimates of both the biomarker and survival processes, as such an analysis ignores the dependence between the repeated longitudinal measurements and survival. Joint modelling of longitudinal and survival data is preferred to separate analyses both to optimally use the available information and to obtain unbiased estimates of parameters describing both processes [8,9]. Joint models are versatile methods for deriving probabilities of survival and forecasts for future levels of biomarkers [10]. There is a long history of the use of joint models of CD4 counts and survival in the HIV literature [7,11–17],among these studies, the most common structure assumed for the association between the longitudinal CD4 count process and survival is a random effects structure, with random intercepts and random slopes for the longitudinal CD4 counts, where survival is regressed on these random effects in the joint model [11,13]. A few have used more flexible structures, including integrated Ornstein–Uhlenbeck processes [18] or splines [15]. Here, following Rizopoulos et al. [19], we consider that different association structures may be better for predicting biomarker and survival processes at different times for different individuals.

A Bayesian approach to joint modeling can be used to derive dynamic individualized predictions of disease progression and survival [6], and to account for the potential for multiple competing association structures to be preferred for prediction at different time points and individuals, Rizopoulos et al. [19] used Bayesian Model Averaging (BMA) to average across dynamic predictions from the competing models. Motivated by the work of Rizopoulos et al. [19], we aim to investigate whether different association structures predict better the longitudinal CD4 count and survival processes for different individuals at different times in their follow-up, for a cohort of people living with HIV who are on anti-retroviral treatment. We derive individual-level predictions of both CD4 count progression and HIV survival, based on a collection of possible models simultaneously, and consider whether combining them using Bayesian model averaging is valuable for this cohort or not.

## Methods

### Data, setting and participants

This study used data from HIV patients from Jimma University Specialized Hospital, Ethiopia, in order to predict the probabilities of HIV-related survival over time by jointly modeling the longitudinal CD4 count representing HIV disease progression and time-to-death processes. While the cohort comprised a total of 854 patients who first received ART between 2009 and 2014, the specific HIV data for this analysis came from a longitudinal study of the subset of 254 patients aged at least 18 years who had at least one measurement of CD4 count. 96 subjects (38%) had only a single CD4 count: given this large proportion of a small sample size, these patients were retained in the analysis population, to maintain power. These patients were followed up for a maximum of 48 months, with visit times at which CD4 counts were recorded occurring approximately every 6 months starting from the first time each of these patients received ART. A histogram of observed CD4 counts in Fig. C1 (Appendix C) shows the data fail to fulfil a normality assumption: logarithmic and square root transformations were considered to achieve normality, but the square-root-transformed CD4 counts appeared more Gaussian than the log-transformed counts (Figs. C2 and C3, Appendix C). In what follows, we therefore use the square root transformation.

### Outcome

The two outcome variables considered for this study were the survival outcome, i.e., time from attendance date to death, measured in months, and the observed CD4 counts, measured in cells/mm^3^ of blood. Of the study population, 13% had died, 16% had transferred their care to another facility, 22% were lost to follow-up and 49% were still under active follow-up, by the end of the study. The World Health Organization (WHO)’s clinical stage of disease [20], the functional status of patients (ambulatory, bedridden or working), age, sex, weight, alcohol use, smoking, drug use and marital status were the covariates available for analysis. The covariate weight is time-dependent and other remaining covariates are fixed at baseline values. The alcohol, smoking and drug use covariates are binary variables for use or not. Further details about the data are found in Temesgen et al. [16].

### Statistical analysis

Since HIV survival is known to be dependent on disease progression, such as measured by CD4 count [7,14,16], careful consideration of the statistical method used to relate the two outcome variables is important. A key characteristic of HIV disease progression is its dynamic nature: the rate of progression is not only different from patient to patient, but also dynamically changes in time for the same patient. Thus, the true potential of the CD4 count biomarker in describing disease progression and its association with survival can only be exploited when repeated measurements of CD4 count are considered in the analysis. The structure of the dependence between the two outcomes is not fully known, and may vary between populations, and even over time within a single patient. To address research questions involving characterization of the association structures between repeated measures and event times, a class of statistical models has been developed known as joint models for longitudinal and time-to-event data [6,21]. Briefly, a mixed effects model is proposed for the longitudinal biomarker observations, of a general form *y_i_* (*t*) = *m_i_* (*t*) + ε_*i*_ (*t*) where the mean *m_i_* (*t*) is the (linear) predictor comprising both fixed and random effects, and ε_*i*_ (*t*) is a normally distributed error term. Simultaneously, a standard survival model is posited for the time-to-event data, regressed both on covariates and on the mean *m_i_* (*t*) of the longitudinal process. Different forms of the regression on *m_i_* (*t*) are possible, including regressing only on the current value of the mean, regressing on both the current value and rate of change, or instead regressing on the random effects that are included in *m_i_* (*t*), for example. Given the random effects in *m_i_* (*t*), both the longitudinal and survival processes are assumed independent, as are the longitudinal responses of each individual. The random effects therefore account both for the association between the longitudinal and the survival outcomes and the correlation between the repeated measurements in the longitudinal process.

Extensions of joint models such as dynamic predictions and accuracy measures have also been implemented [15,19,22]. Dynamic prediction is a method for updating predictions ahead in time of both the longitudinal and survival processes, whenever a new measurement of the longitudinal biomarker is taken. Here three joint models of the evolution of the CD4 count process and HIV survival are fitted to the data in a Bayesian framework, each with a different association structure. Dynamic predictions are derived from each of the three models and are combined using Bayesian model averaging [19,23]. The Bayesian approach to joint modelling [24,25] was implemented using the JMbayes package in R version 1.2.5033 that implements both the joint models and the Bayesian model averaging. Full details on the formulation of these joint models and dynamic prediction can be found in Appendix A, but are briefly summarized below.

**Fig. 1:**
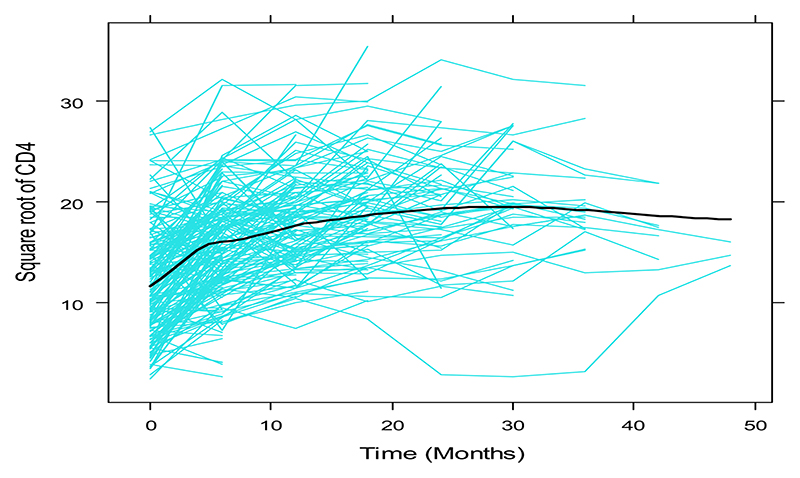
Individual and mean profiles of observed CD4 count data over time.

**Fig. 2:**
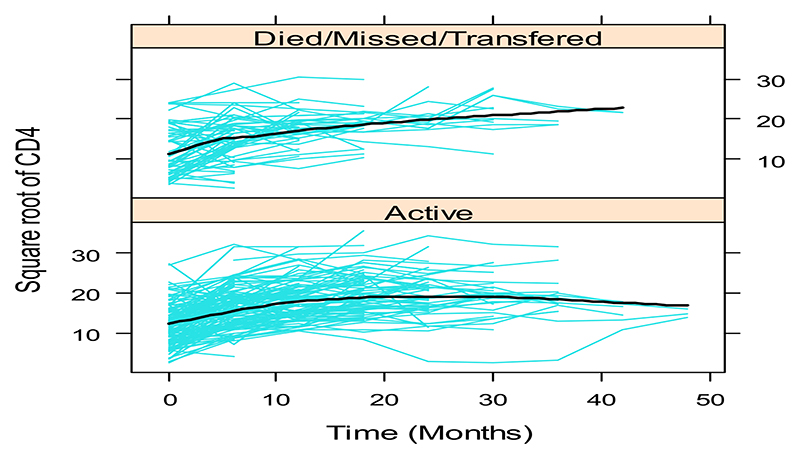
Individual and mean profiles of square root CD4 counts over time categorized by status.

In a preliminary step, covariate selection for the sub-model for the longitudinal square-root CD4 process was carried out, fitting linear mixed models in a frequentist framework and using likelihood ratio tests, Bayesian Information Criterion (BIC) and Akaike’s Information Criterion (AIC) to choose between models (results not shown). The chosen sub-model included a flexible specification of the subject-specific square-root CD4 trajectories, using natural cubic splines of time to capture the non-linear trajectories (Figs. 1; 2). In addition to the spline of time, significant covariates retained in the linear mixed model were the functional status of patients (ambulatory, bedridden or working) and alcohol consumption, with an interaction between functional status and the spline of time:

$$\begin{array}{cc} y_{i}(t)= & m_{i}(t)+\varepsilon_{i}(t)\\ \;\;\;\;\;\;\;\;\;\;\;\;= & \beta_{0}+\beta_{1}FNS_{i}+\beta_{2}{\;\text{Alcohol}\;}_{i}+\beta_{3}VT_{i}*FNS_{i}+\\ & +\beta_{4}B_{1}(t)+\beta_{5}B_{2}(t)+\\ & +b_{i0}+b_{i1}B_{1}(t)+b_{i2}B_{2}(t)+\varepsilon_{i}(t) \end{array}$$

where *y_i_* (*t*) is the square-root CD4 count for individual *i* at time *t*, ***B***_*n*_(*t*) for *n* = 1, 2 denotes the B-spline basis for a natural cubic spline with 2 degrees of freedom; FNS and Alcohol denote the variables functional status and alcohol intake respectively; and the term VT* FNS denotes the interaction between the 2df spline of patient visit times and functional status. The β‘s are fixed effects whereas the *b*’*s* are random effects. For the survival process, after an initial covariate selection step which resulted in functional status, alcohol intake, marital status and weight as significant covariates for survival, we consider three relative risk models, each positing a different association structure between the two processes, namely:

$$\begin{array}{cc} M_{1}(t):h_{1}(t)= & h_{0}(t)\exp\left\{\gamma_{1}FNS_{i}+\gamma_{2}{\;\text{Alcohol}\;}_{i}+\gamma_{3}MS_{i}+\gamma_{4}wt_{i}+\alpha_{1}m_{i}(t)\right\},\\ M_{2}(t):h_{2}(t)= & h_{0}(t)\exp\left\{\gamma_{1}FNS_{i}+\gamma_{2}{\;\text{Alcohol}\;}_{i}+\gamma_{3}MS_{i}+\gamma_{4}wt_{i}+\alpha_{1}m_{i}(t)+\alpha_{2}m_{i}^{\prime }(t)\right\}\\ \;\text{with}\;{m^{\prime }}_{i}(t)= & d\left\{x_{i}^{T}(t)\beta +Z_{i}^{T}(t)b_{i}\right\}/dt\\ M_{3}(t):h_{3}(t)= & h_{0}(t)\exp\left\{\gamma_{1}FNS_{i}+\gamma_{2}{\;\text{Alcohol}\;}_{i}+\gamma_{3}MS_{i}+\gamma_{4}wt_{i}+\alpha_{1}b_{i0}+\alpha_{2}b_{i1}+\alpha_{3}b_{i2}\right\} \end{array}$$

where MS and wt are the marital status and weight variables; the baseline hazard *h*_0_(*t*) is approximated with splines (see Eq. (A3) of Appendix A), *m_i_*(*t*) is the current true value of the CD4 count trajectory, *m′_i_*(*t*) is the slope of the trajectories at time t (rate of change in CD4 count), *γ* are regression parameters of the survival model and *α* are parameters describing the strength of the association between the CD4 count and survival processes.

### Individualized dynamic predictions

Based on each joint model, prediction of survival probabilities and future CD4 counts for a new individual *j* who has a set of longitudinal square-root CD4 counts Y_*j*_(*t*) = { *y_j_*(*s*);0 ≤ *s* ≤ *t*} and a vector of baseline covariates *w_j_* is required. For any time *u*>*t*, the focus of interest is in predicting both the conditional probability *π_j_*(*u*|*t*) that subject *j* will survive at least up to *u* and his/her predicted CD4 count at *u*. At each time of interest (e.g. a clinic visit time) *t’*, *t* < *t′* < *u*, these predictions are dynamically updated, as extra information is recorded for the patient. That is, the prediction *ω_j_*(*u*|*t*) of the square-root CD4 count *y_i_*(*u*) that is based on the information available up to time *t*, can be updated at time *t′*, to produce a new prediction *ω_j_*(*u*|*t*′) that uses the additional longitudinal information up to the latter time point *t’*, Under the Bayesian joint modelling framework, both predictions *π_j_*(*u*|*t*) and *ω_j_*(*u*|*t*) are based on the posterior predictive distribution, as given in Appendix A.

Standard model selection techniques for choosing between the three association structures may prefer different models, depending which model selection criteria is used [26,27], particularly in contexts where different association structures may produce better predictions for different individuals at different time points. BMA [19,22] explicitly takes into account model uncertainty by applying Bayesian inference to model selection. Each model is given a prior weight, in this case assuming each association structure is equally likely, and the resulting posterior model weights are used to average over the estimates. Here, following Rizopoulos et al., instead of averaging estimates over the association structures, the predictions *π_j_*(*u*|*t*) and *ω_j_*(*u*|*t*) are averaged over the different association structures. In some contexts, this BMA approach can produce less risky predictions via a straightforward model choice criteria [28].

## Results

### Descriptive summaries

Figs. 1 and 2 show that some individual longitudinal CD4 count trajectories had strong nonlinear patterns, motivating the flexible structure of the joint models proposed in Section 2

A total of 254 subjects (of which 54% male and 46% female) were observed in the dataset with a median age of 30 years (interquartile range, IQR, 26–38). The individual follow-up times ranged from 0 to 48 months. The median baseline CD4 count of the subjects included in the analysis was 129 cells/mm3 (IQR 61–247 cells/mm3). The square-root transformed CD4 count of the subjects ranged from 2.5 to 27.4 with a first quartile of 7.8, a median of 11.4 and a third quartile of 15.7.

Table B1 (Appendix B) summarizes the demographics of the study population. Based on the functional status of subjects, 368/792(46%) were able to work, 376/792(47%) were ambulatory, and 48/792(6%) were bedridden. Around 277/792(35%) drink alcohol, while 515/792(65%) had no history of alcohol consumption. 341/792(43%) of individuals were married, while 53/792(7%) were divorced.

### Parameter estimates from the three joint models

Table 1 shows posterior mean estimates and their corresponding 95% credible intervals for the parameters in the longitudinal and survival sub-models for each of the three different association structures. In the models including current CD4 count in the association structure (Models 1 and 2), the association parameters indicate that HIV survival at any time *t* is not significantly associated with the current underlying CD4 count at the same time point, whereas the rate of change in CD4 count over time is significantly associated with HIV survival (Models 2 and 3). The parameter estimates in the relative risk models and in the linear mixed models show slight variability between the posited association structures. Model 3, having an association structure which assumes only the random effects are shared between the two processes, has the lowest Deviance Information Criterion (DIC) where a lower DIC implies a better fitting model relative to models with higher DIC. In contrast, in the Bayesian model-averaged predictions, model 1, where the subject-specific linear predictor from the mixed model (current value of CD4 count) is included as a time-varying covariate in the relative risk model is the only model with almost all non-zero posterior weights for all time points for each individual (Table B2, Appendix B). The following table (Table 2) shows the 12 patients who have non-zero posterior weights for Model 2 and Model 3 at some time points. Given the preference of the model averaging approach for Model 1 in predicting CD4 counts and HIV survival, for the majority of patients, we interpret here only the parameter estimates for Model 1.

Convergence of the Markov chain Monte Carlo algorithm for this selected Model 1 is demonstrated in Appendix C (Fig. C4). The Model 1 regression coefficient estimates for the CD4 count process suggest that subjects whose functional status was working were associated with a higher CD4 count (*β* =1.84; 95% CrI: 0.10, 3.51) whereas those whose functional status was bedridden were associated with lower CD4 count (*β* =−3.09; 95% CI: –5.07, –1.05), compared with the baseline ambulatory functional status group. The cubic spline of visit times had positive effects on the square-root CD4 measurements, indicating increasing CD4 counts with time, and interacted significantly with functional status. Functional status, alcohol consumption and weight also had substantial effects on the hazard of HIV mortality: hazard ratio 0.47 for patients able to work and 2.11 for patients who are bedridden compared to those with ambulatory functional status; 1.64 times higher for patients who consume alcohol compared to the non-alcohol drinking group; and hazard ratio 0.98 for each unit increase in weight. A unit increment in the patient-specific current square-root CD4 count decreases the hazard of HIV mortality 0.98 times (*β* = −0.02; 95% CrI: –0.07, 0.03). The posterior probability which is close to zero for the association between current CD4 count and mortality may be due to the inclusion of functional status in both the CD4 count process model and the survival model: perhaps this covariate, since it is associated with CD4 count and has interaction with time, already adequately accounts for the time-varying association of health with mortality

### Model-averaged predictions

**Fig. 3:**
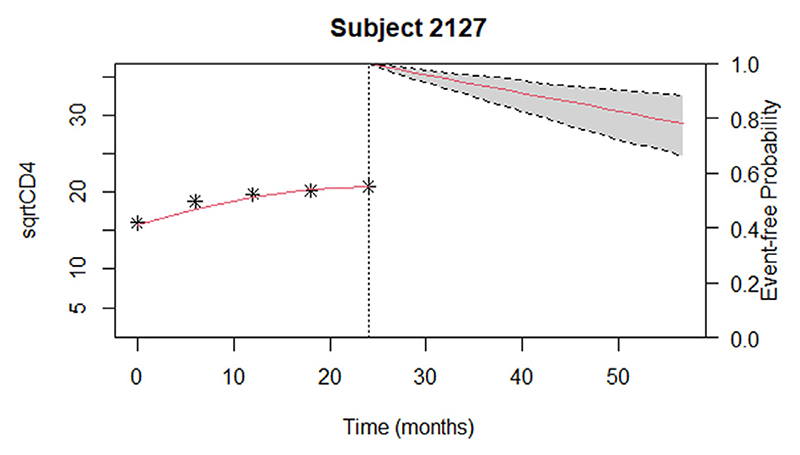
Observed (stars) and estimated (red line to left of vertical dotted line) CD4 count up till time of last observed CD4 count; and survival probability predicted at time of last observed CD4 count for patient 2127.

**Fig. 4:**
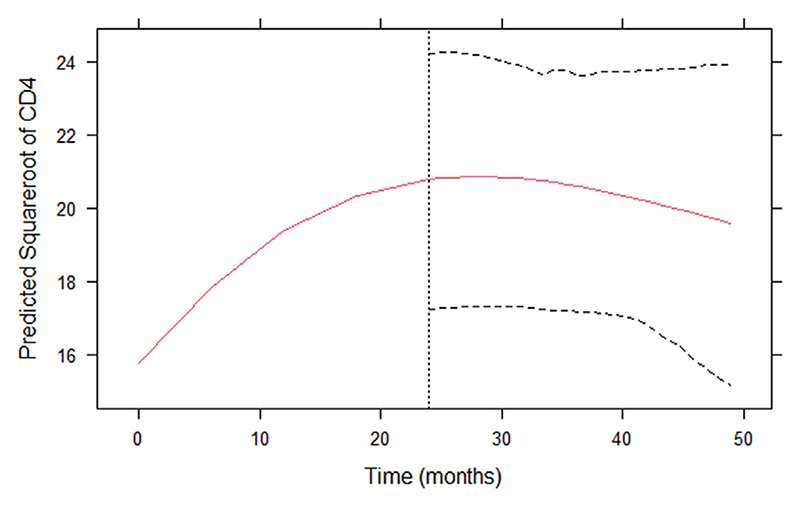
Estimated CD4 count trajectory (left of vertical dotted line) and dynamic prediction of CD4 count made at time of last observed CD4 count (right of vertical dotted line) for patient 2127.

Table 2 shows model-averaged predictions for twelve individuals, who have non-zero posterior weights for Model 2 and Model 3 at some time points. The table also shows the time-dependent subject-specific BMA weights for the three joint models. The dynamic predictions are updated at each visit time for each patient, using the CD4 counts observed up to the current visit time to predict both future CD4 count trajectories and HIV survival. As noted, only Model 1 (which considers the current estimated value of the CD4 count trajectory) has non-zero posterior weight at almost all time points. Figs. 3 and 4 display these averaged predictions for one example patient (id 2127), who has non-zero posterior weights for model 1. This patient’s observed and expected CD4 counts increase over the study period.

The vertical dotted lines in both Figs. 3 and 4 represent the time point of the last square-root CD4 count observation. The left sides of the panels present the observed square root of CD4 counts and the right shows the predicted survival (Fig. 3) and predicted longitudinal trajectory of CD4 count for subject 2127. The dashed lines represent the corresponding 95% point wise posterior predictive intervals. The dynamic survival probabilities and dynamic predictions of CD4 counts for patients 2769 and 3790, who have non-zero posterior weights for model 3 and model 2 respectively, are given in Appendix C (Fig. C5–C8).

## Discussion

We have fitted three joint models incorporating different association structures that relate HIV survival to the longitudinal trajectories of patient CD4 counts; then used Bayesian model averaging techniques to base dynamic predictions on the best model for each individual and time point respectively. The model averaging allows for a precision medicine approach to individual prediction, by allowing for robustness to model misspecification. For most patients in our study, Model 1, which considers the current estimated value of the CD4 count trajectory, was the only model with non-zero posterior weight at most time points. But a notable proportion (12/254 = 0.047) of patients had visit times at which either Model 2 or Model 3 was preferred for predicting future CD4 counts and survival. Despite this preference for other models a different time points, there is not much difference in prediction using individual models versus the averaging method in our data set. This small magnitude of differences suggests robustness of the predictions to alternative association structures (Table B3 in the Appendix) for this study.

The estimates of the association parameter in Model 1, together with the dynamic survival probability plots, showed that a unit increase in the CD4 count decreases the hazard of HIV mortality, indicating that those with higher CD4 count survive longer. The association parameter has close to zero posterior probability, however, suggesting that other covariates in the survival model already adequately explained the variation in mortality. In particular, since the posterior probability for the association of functional status with both CD4 count and survival is a value close to one, as well as the interaction with time in the CD4 count sub-model, perhaps there is some collinearity between functional status and CD4 count which lead to the lack of significant association with survival. Note that we found an increase in the width of the prediction intervals for the future CD4 counts as time progressed. Note also that Rizopoulos et al. [22] state that differences in prediction performance between different specifications of the association structure might be due to the dependence structure not being correctly specified. Rizopoulos et al. [19] compared dynamic prediction results from five joint models with different parameterizations in the survival sub-model and found that the predicted conditional survival probabilities showed considerable variability between the six parameterizations. As a response to the challenge of different results from different parameterizations, Barrett & Su [15] use a different approach, using a flexible non- or semi-parametric specification of the association structure, rather than a parametric specification, to avoid mis-specifying the dependence structure. Although BMA has some interesting features, it has some potential drawbacks as well, including being computationally intensive, since it requires fitting all the models we wish to average. Other approaches to individualized dynamic prediction include stacking and pseudo-BMA [29] and land marking [30]. A number of other papers have also compared prediction models for dynamic prediction [31–33]. This study is limited to a single university hospital; it will of interest to apply the method more widely to data from different hospitals throughout the country.

The main aim of this study was to investigate how dynamic predictions from joint models incorporating different association structures can be combined using Bayesian model averaging, and whether BMA is useful for our particular cohort. The result from this study showed that there is some variability between the three association parameterizations we used in terms of parameter estimates. Many studies use standard likelihood information criteria to decide on the selection of appropriate joint model and make predictions. However, we found that in terms of model fit (balanced by a measure of model complexity), model 3 was preferred; whereas in terms of dynamic prediction, the BMA approach showed model 1 was preferred for the majority of patients. For the subset of 12 patients for whom there was non-zero posterior probability of either model 2 or 3 at different time points, we found that BMA gave almost the same dynamic predictions to those obtained from a single model (Model 1) alone. Even if the averaged predictions are not that different from model-specific predictions in general in this particular dataset, as also recognized in other applications [19], a single prognostic model may not be adequate for all patients at all times, in which case BMA may provide a good solution to sensitivity of predictions to model specification, accounting for model uncertainty. Investigation of different parameterizations and sensitivity of parameter estimates and predictions to model specification is essential to the provision of individual-level predictions of patient progression and survival, i.e. to a precision medicine approach.

## Supplementary Material

Supplementary Appendix

## Figures and Tables

**Table 1:** Parameter estimates (posterior mean), 95% credible intervals (CrI, in brackets), two-sided posterior probability (tail area) that the parameter is more extreme than 0 (i.e. *p* = 2 × min{Pr(θ > 0), Pr(θ < 0)} for each parameter θ, in square brackets) and model comparison results (Deviance Information Criterion, DIC) from the three joint models fitted to the HIV data.

**Parameter**			*M*_1_(*t*)	*M*_2_(*t*)	*M*_3_(*t*)
Survival process	Functional status: bedridden		0.75 (0.19, 1.28)[0.010]	0.89 (0.26, 1.43)[0.009]	1.09 (0.39, 1.98)[**<**0.001]
	Functional status: working		−0.76 (−1.17, −0.33)[**<**0.001]	−0.65 (−1.09, −0.22)[0.001]	−1.14 (−1.87, −0.53)[**<**0.001]
	Alcohol intake: yes		0.49 (0.11, 0.87)[0.007]	0.49 (0.10, 0.89)[0.011]	0.70 (0.22, 1.22)[**<**0.001]
	Weight		−0.02 (−0.04, −0.00)[0.020]	−0.02 (−0.04, −0.001)[0.029]	−0.02 (−0.06, 0.001)[0.052]
	Marital status: married		−0.38 (−0.92, 0.23)[0.234]	−0.46 (−1.07, 0.10)[0.117]	−0.38 (−1.25, 0.49) [0.384]
	Marital status: separated		0.22 (−0.48, 0.92)[0.553]	0.18 (−0.52, 0.86)[0.593]	0.22 (−0.75, 1.23)[0.634]
	Marital status: single		−0.44 (−1.02, 0.18)[0.167]	−0.50 (−1.09, 0.11)[0.101]	−0.61 (−0.1.69, 0.35)[0.203]
	Marital status: widowed		−0.92 (−2.03, 0.05)[0.058]	−0.86 (−1.85, 0.85)[0.073]	−0.98 (−2.38, 0.35)[0.133]
	Association parameter (mean of longitudinal process) α1		−0.02 (−0.07, 0.03)[0.452]	−0.003 (−0.05, 0.05)[0.914]	
	Association parameter (slope of longitudinal process) α2			1.74 (0.22, 3.37)[0.025]	
	Association parameter (random effect for spline intercept) α1				0.17 (0.12, 0.24)[**<**0.001]
	Association parameter (random effect for spline basis 1 of visit time) α 2				0.04 (−0.001, 0.09)[0.064]
	Association parameter (random effect for spline basis 2 of visit time) α 3				0.77 (0.32, 1.34)[**<**0.001]
Longitudinal process	Fixed effects	Intercept	11.55 (10.71,12.39)[**<**0.001]	11.57 (10.76, 12.38)[**<**0.001]	11.54 (10.71,12.43)[**<**0.001]
		Visit time: spline basis 1	15.46 (13.72,17.17)[**<**0.001]	15.93 (14.19, 17.62)[**<**0.001]	15.36 (13.66,17.16)[**<**0.001]
		Visit time: spline basis 2	1.69 (1.13, 2.29)[**<**0.001]	1.67 (1.13, 2.21)[**<**0.001]	1.76 (1.32, 2.28)[**<**0.001]
		Functional status: bedridden	−3.09 (−5.07, −1.05)[0.003]	−3.18 (−5.02, −1.20)[0.001]	−3.14 (−5.17, −1.15)[0.003]
		Functional status: working	1.84 (0.10, 3.52)[0.035]	1.79 (0.19, 3.46)[0.031]	1.90 (0.29, 3.60)[0.016]
		Alcohol intake: yes	−0.61 (−1.49, 0.29) [0.177]	−0.46 (−1.32, 0.43)[0.297]	−0.64 (−1.52, 0.21)[0.143]
		Visit time: spline basis 1* Functional status: bedridden	−0.12 (−4.30, 4.19)[0.965]	0.005 (−3.94, 4.13)[0.997]	0.0003 (−4.13, 4.20)[0.983]
		Visit time: spline basis 2* Functional status: bedridden	4.67 (3.34, 6.04)[**<**0.001]	4.71 (3.43, 6.04)[**<**0.001]	4.66 (3.49, 5.77)[**<**0.001]
		Visit time: spline basis 1* Functional status: working	−6.03 (−8.51, −3.51)[**<**0.001]	−6.44 (−9.03, −3.90)[**<**0.001]	−6.04 (−8.69, −3.47)[**<**0.001]
		Visit time: spline basis 2* Functional status: working	0.99 (0.19, 1.79)[0.022]	1.01 (0.28, 1.76)[0.018]	0.94 (0.19, 1.60)[0.020]
	Random	Intercept	4.22	4.18	4.23
	effects	Visit time: spline basis 1	7.92	7.55	7.74
	standard	Visit time: spline basis 2	2.38	2.26	2.02
DIC	deviation		9696.12	9675.095	9670.78

**Table 2:** BMA posterior weights for patients with non-zero posterior weights for model 2 and model 3, with their marginal density of the data given model M_k_ for each model.

Subject	Months	Square root of CD4	M1	M2	M3
996	0	22.1	1.00	0.00	0.00
	6	24.2	1.00	0.00	0.00
	12	24.2	1.00	0.00	0.00
	18	23.2	0.00	1.00	0.00
	0	3.7	1.00	0.00	1.00
2161	6	13.5	1.00	0.00	0.00
	12	23.6	1.00	0.00	0.00
	24	21.3	1.00	0.00	0.00
	30	19.8	0.04	0.96	0.00
	36	19	0.00	1.00	0.00
	0	17.4	1.00	0.00	0.00
2204	6	20.1	1.00	0.00	0.00
	12	24.8	0.99	0.00	0.00
	18	26.6	1.00	0.00	0.00
	24	23.7	0.00	1.00	0.00
	0	16.8	1.00	0.00	0.00
2248	6	19	1.00	0.00	0.00
	12	13.5	1.00	0.00	0.00
	18	17.9	1.00	0.00	0.00
	24	15.4	0.00	1.00	0.00
2769	0	26.9	1.00	0.00	0.00
	6	32.1	0.99	0.00	0.00
	12	28.1	0.00	0.00	1.00
	18	29.5	1.00	0.00	0.00
	24	27.9	0.01	0.00	0.99
2877	0	16	1.00	0.00	0.00
	6	22	1.00	0.00	0.00
	12	23.3	1.00	0.00	0.00
	18	26.3	1.00	0.00	0.00
	24	25	1.00	0.00	0.00
	30	27.6	0.00	1.00	0.00
2937	0	10.7	1.00	0.00	0.00
	6	22.9	1.00	0.00	0.00
	12	16.1	1.00	0.00	0.00
	18	18.4	1.00	0.00	0.00
	24	28	0.00	1.00	0.00
3137	0	18	1.00	0.00	0.00
	6	20.5	1.00	0.00	0.00
	12	23.6	0.99	0.00	0.00
	18	35.4	0.00	1.00	0.00
3236	0	22.7	1.00	0.00	0.00
	6	14.8	0.99	0.00	0.00
	12	19.1	0.99	0.00	0.00
	18	23.9	1.00	0.00	0.00
	24	21.7	0.00	1.00	0.00
3758	0	22.2	0.18	0.82	0.00
3790	0	21	1.00	0.00	0.00
	6	17.5	1.00	0.00	0.00
	12	20.9	1.00	0.00	0.00
	18	18	1.00	0.00	0.00
	24	17.3	1.00	0.00	0.00
	30	15.7	1.00	0.00	0.00
	36	19.9	1.00	0.00	0.00
	42	17.5	0.08	0.92	0.00
3840	0	15.3	1.00	0.00	0.00
	6	14.9	0.00	1.00	0.00
	Marginal density		–2828.23	–2839.41	2845.85

## Data Availability

The datasets analysed in this study are not publicly available due to patient confidentiality, but are available from the corresponding author on reasonable request.

## Abbreviations

AIC: Akaike information criterion
AIDS: Acquired Immune Deficiency Syndrome
ART: Antiretroviral therapy
BIC: Bayesian information criterion
BMA: Bayesian Model Averaging
CD4: cluster of differentiation 4
EDHS: Ethiopian Demographic and Health Survey
HIV: Human Immunodeficiency Virus
IQR: Interquartile range
MCMC: Markov chain Monte Carlo
PLHIV: People living with HIV
SNNPR: Southern Nations Nationalities and Peoples’ Region
TB: Tuberculosis
UNAIDS: United Nations Acquired Immune Deficiency Syndrome
WHO: World Health Organization
