# Supplementary material for: Dynamic predictions from longitudinal CD4 count measures and time to death of HIV/AIDS patients using a Bayesian joint model
Source: Sci Afr. Author manuscript; Available in PMC 2023 Mar 1. (PMC7614071; doi:10.1016/j.sciaf.2022.e01519)
Supplement: Supplementary Appendix [file EMS159254-supplement-Supplementary_Appendix.pdf]

Appendix A: Models and formulas

Joint Models for Longitudinal and Time to Event Data

Model Specification

We denote T_i^*as a true event time for the ith subject, T_i the observed event time, defined as the minimum of the potential censoring time C_i and T_i^*, and by $\delta_i = \mathrm{I}(T_i^* < C_i)$ the event indicator. A standard approach is the Cox model (Cox, 1972) [34], postulating a relative risk model of the form

$$h_i(t|\mathrm{M}_i(t), w_i) = \lim_{dt \to 0} \Pr\{t \le T_i^* < t + dt | T_i^* \ge t, \mathrm{M}_i(t), w_i\}/dt = h_0(t)\exp\left\{\gamma^T w_i + \alpha m_i(t)\right\}, t > 0, \quad \text{(A1)}$$

where $\mathrm{M}_i(t) = \{m_i(s), 0 \le s < t\}$ denotes the history of the true unobserved longitudinal processes up to time point t, $h_o(.)$ denotes the baseline hazard or risk function, and w_i is a vector of baseline covariates with a corresponding vector of regression coefficients γ. Similarly, parameter α quantifies the effect of the underlying longitudinal outcome on the risk of

an event. To complete the specification of Eq. (1), we need to define the baseline risk function$h_0(.)$. A standard option is to use a risk function corresponding to a known parameter distribution. Under the piecewise-constant model, the baseline risk function takes the form:

$$h_0(t) = \sum_{q=1}^{Q} \xi_q \mathrm{I}\left(\nu_{q-1} < t < \nu_q\right), \tag{A2}$$

where $\nu_0 < \nu_1 < .... < \nu_Q$ denotes knots splitting the time scale, with ν_Q being larger than the largest observed time, and ξ_q denotes the value of the hazard in the interval $(\nu_{q-1}, \nu_q]$. As the number of the knots increases the specification of the baseline hazard becomes more flexible. The log baseline risk function log h_0 (t) is extended to B-spline basis functions for cubic splines for the regression splines model as follows:

$$\log h_0(t) = k_0 + \sum_{d=1}^{m} k_d B_d(t, q), \tag{A3}$$

Where $k^T = (k_0, k_1, \ldots, k_m)$ are the spline coefficients, q denotes the degree of the B-splines basis functions B(.), and $m = \ddot{m} + q - 1$,with $\ddot{m}$ denoting the number of interior knots. According to the piecewise-constant model, increasing the number of knots increases the approximate $h_0(.)$ flexibility. However, in both approaches, we need to keep a balance between bias and variance and avoid over fitting.

Laird and Ware, 1982 [35]; Harville, 1997 [36]; and Verbeke and Molenberghs, 2000 [37] used linear mixed models to model the longitudinal process. In linear mixed models, the observed longitudinal data consist of the measurements:

$$\begin{aligned} &y_i(t) = \mathrm{X}_i^T(t)\beta + \mathrm{Z}_i^T(t)b_i + \varepsilon_i(t) \\ &b_i \sim \mathrm{N}(0, C), \qquad \varepsilon_i(t) \sim \mathrm{N}(0, \sigma^2) \end{aligned} \tag{A4}$$

where $y_i(t)$ denotes the value of the longitudinal outcome at any particular time point t, x_i (t) and z_i (t) denote the time-dependent design vectors for the fixed-effects β and for the random effects b_i, respectively. C is the variance-covariance matrix for the random effects and $\varepsilon_i(t)$ is the corresponding error terms that are assumed independent of the random effects with mean zero and variance σ^2.

Estimation of joint models

The main estimation method proposed for joint models is (semi-parametric) maximum likelihood (Wolfsohn and Tsiatis, 1997 [38]; Henderson et al., 2000 [21]; Hsieh et al., 2006 [39]). Bayesian estimation of joint models using MCMC techniques has been employed by Rizopoulos et al. (2014) [19] whereas Barrett and Su (2017) [15] used maximum penalized likelihood approach that maximizes the likelihood function corresponding to the joint distribution of the longitudinal and time-to-event outcomes. Under the Bayesian approach, estimation of the joint model's parameters proceeds using Markov chain Monte Carlo (MCMC) algorithms. The expression for the posterior distribution of the model parameters is derived under the assumptions that given the random effects (b_i), both the longitudinal and event time process are assumed independent, and the longitudinal responses of each subject are assumed independent. This means that the random effects account both for the association between the longitudinal and the event outcomes, and the correlation between the repeated measurements in the longitudinal process. Formally, we have

$$p(y_i, T_i, \delta_i | b_i; \theta) = p(T_i, \delta_i | b_i; \theta) p(y_i | b_i; \theta), \text{ and} \tag{A5}$$

$$p(y_i | b_i; \theta) = \prod_l p\{y_i(t_{il}) | b_i; \theta\} \tag{A6}$$

where $\theta = (\theta_t^T, \theta_y^T, \theta_b^T)^T$ denotes the full parameter vector, with θ_t denoting the parameters for the event time outcome, θ_y the parameters for the longitudinal outcomes and θ_b the unique parameters of the random effects covariance matrix, and y_i is the $n_i x1$ vector of longitudinal responses of the ith subject. And p(.) denotes an appropriate probability density function. Under these assumptions the posterior distribution is analogous to:

$$p(\theta, b) \propto \prod_{i=1}^{n} \prod_{l=1}^{n_i} p(y_{il} | b_i, \theta) p(T_i, \delta_i | b_i, \theta) p(b_i | \theta) p(\theta) \tag{A7}$$

where

$$p(y_{il} | b_i, \theta) = \exp\{[y_{il}\psi_{il}(b_i) - c\{\psi_{il}(b_i)\}]/a(\phi) - d(y_{il}, \phi)\}$$

With $\psi_{il}(b_i)$ and ϕ denoting the natural and dispersion parameters in the exponential family, respectively, c(·), a(·), and d(·) are known functions specifying the member of the exponential family, and for the survival part

$$p(T_i, \delta_i | b_i, \theta) = h_i(T_i | \mathrm{H}_i(T_i))^{\delta_i} \exp\left\{ -\int_0^{T_i} h_i(s | \mathrm{H}_i(s)\, ds) ds \right\} \tag{A8}$$

With h_i (.) given by (1).The integral in the definition of survival function

$$S_I(t|\mathrm{H}_i(t), w_i(t)) = \exp\left\{-\int_0^t h_0(s)\exp\left[\gamma^T w_i(t) + f\{\mathrm{H}_i(s), \alpha\}\right]ds\right\} \tag{A9}$$

does not have a closed-form solution, and thus a numerical method must be employed for its evaluation. Standard options are the Gauss-Kronrod and Gauss-Legendre quadrature rules. For the parameters θ we take standard prior distributions. In particular, for the vector of fixed effects of the longitudinal submodel β, for the regression parameters of the survival model γ, for the vector of spline coefficients for the baseline hazard γ_{h_0}, and for the association parameter α we use independent univariate diffuse normal priors. The penalized version of the B-spline approximation to the baseline hazard can be fitted by specifying for γ_{h_0} the prior (Lang and Brezger 2004) [40]:

$$p\left(\gamma_{h_0}|\tau_h\right) \propto \tau_h^{p(k)/2}\exp\left(-\frac{\tau_h}{2}\gamma_{h_0}^T K\gamma_{h_0}\right) \tag{A10}$$

where τ_h is the smoothing parameter that takes a Gamma(1, 0.005) hyper-prior in order to ensure a proper posterior for γ_{h_0}, $K = \Delta_r^T\Delta_r + 10^{-6}I$, where Δ_r denotes the rth difference penalty matrix, and ρ(K) denotes the rank of K. For the covariance matrix of the random effects we assume an inverse Wishart prior, and when fitting a joint model with a normally distributed longitudinal outcome, we take an inverse-Gamma prior for the variance of the error terms $\sigma\ ^2$.

Model Selection Techniques

When interest is in comparing non-nested models, information criteria are typically used. The two most commonly used information criteria are the Akaike's Information Criteria (AIC; Akaike, 1974) [26] and the Bayesian Information Criteria (BIC; Schwarz, 1978) [27] (Figs. C6, C7).

$$AIC = -2\ell\left(\hat{\theta}\right) + 2p \tag{A11}$$

$$BIC = -2\ell\left(\hat{\theta}\right) + p\log(n) \tag{A12}$$

where p denotes the number of parameters in the model and n denote the number of observations.

We focus here on dynamic BMA predictions of survival probabilities. BMA predictions for the longitudinal outcome can be produced with similar methodology. Here we assume that we have available data $D_n = \{T_i, \delta_i, y_i; i = 1, \ldots, n\}$ based on which we fit $M_1, \ldots, M_K$joint models with different association structures. Interest is in calculating predictions for a new subject j from the same population who has provided a set of longitudinal measurements $Y_j(t)$, and has a vector of baseline covariates w_j . We let $D_j(t) = \{T_J^* > t, Y_j(t), w_t\}$ denote the available data for this subject. The model-averaged probability of subject j surviving time $u >$ t, given survival up to t is given by the expression:

$$\Pr\left(T_j^* > u\middle|D_j(t), D_n\right) = \sum_{k=1}^{K}\Pr\left(T_j^* > u\middle|M_k, D_j(t), D_n\right)p\left(M_k\middle|D_j(t), D_n\right) \tag{A13}$$

The first term in the right-hand side of Eq. (14) denotes the model-specific survival, while the second denotes the posterior weights of each competing joint models. For calculating the model weights, we observe that these are written as (Rizopoulos et al. 2014)[19]:

$$p\left(M_k\middle|D_j(t), D_n\right) = \frac{p\left(D_j(t)|M_k\right)p(D_n|M_k)p(M_k)}{\sum_{l=1}^{k} p\left(D_j(t), |M_l\right)p(D_n|M_l)p(M_l)} \tag{A14}$$

where

$$p\left(D_j(t)|M_k\right) = \int p\left(D_j(t)\middle|\tilde{\theta}_k\right)p\left(\tilde{\theta}_k|M_k\right)d\tilde{\theta}_k$$

With $\tilde{\theta}_k^t = (\theta_K^T, b_j^T)$and $p(D_n|M_k)$ is defined analogously. The likelihood part $p(D_n|\theta_k)$ is based on (3), and Similarly

$$p\left(D_j(t)|\theta_k\right) = p\left(\mathrm{Y}_j(t)\middle|b_j, \theta_k\right)S_j\left(t\middle|b_j, \theta_k\right)p\left(b_j|\theta_k\right) \tag{A15}$$

Thus, the subject-specific information in the model weights at time t comes from the available longitudinal measurements $p(\mathrm{Y}_j(t)$ but also from the fact that this subject has survived up till this time.

Appendix B: Tables

Table B1
Demographic and clinical characteristics of the study subjects.

Variable		Number (%)
Sex	Male	429(54)
	Female	363(46)
FNS	Ambulatory	376 (47)
	Bedridden	48 (6)
	Working	368 (46)
Marital status	Divorced	53 (7)
	Married	341 (43)
	Separated	82 (10)
	Single	253 (32)
	Widowed	63 (8)
Alcohol	No	515 (65)
	Yes	277 (35)
Survival Status	Active	126(49)
	Died	32(13)
	Missed	56(22)
	Transferred	40(16)

Table B2
BMA posterior weights for predictions at the final visit time with the three joint models for all subjects, averaged over their visit times, with their marginal density of the data given model M_k for each model in the final row.

ID	M1	M2	M3	ID	M1	M2	M3	ID	M1	M2	M3
362	1.00	0.00	0.00	3066	1.00	0.00	0.00	3740	1.00	0.00	0.00
996	0.00	1.00	0.00	3094	1.00	0.00	0.00	3741	1.00	0.00	0.00
2116	1.00	0.00	0.00	3100	1.00	0.00	0.00	3748	1.00	0.00	0.00
2120	1.00	0.00	0.00	3102	1.00	0.00	0.00	3753	1.00	0.00	0.00
2127	1.00	0.00	0.00	3108	1.00	0.00	0.00	3758	0.18	0.82	0.00
2142	1.00	0.00	0.00	3117	1.00	0.00	0.00	3763	1.00	0.00	0.00
2146	1.00	0.00	0.00	3118	1.00	0.00	0.00	3764	1.00	0.00	0.00
2151	1.00	0.00	0.00	3122	1.00	0.00	0.00	3765	1.00	0.00	0.00
2161	0.00	1.00	0.00	3134	1.00	0.00	0.00	3766	1.00	0.00	0.00
2204	0.00	1.00	0.00	3137	0.00	1.00	0.00	3773	1.00	0.00	0.00
2211	1.00	0.00	0.00	3156	1.00	0.00	0.00	3774	1.00	0.00	0.00
2235	1.00	0.00	0.00	3192	1.00	0.00	0.00	3777	1.00	0.00	0.00
2236	0.97	0.03	0.00	3223	1.00	0.00	0.00	3780	1.00	0.00	0.00
2242	1.00	0.00	0.00	3236	0.00	1.00	0.00	3783	1.00	0.00	0.00
2248	0.00	1.00	0.00	3240	1.00	0.00	0.00	3790	0.08	0.92	0.00
2251	1.00	0.00	0.00	3265	1.00	0.00	0.00	3801	1.00	0.00	0.00
2252	1.00	0.00	0.00	3273	1.00	0.00	0.00	3803	1.00	0.00	0.00
2266	0.98	0.02	0.00	3275	1.00	0.00	0.00	3805	1.00	0.00	0.00
2267	1.00	0.00	0.00	3278	1.00	0.00	0.00	3806	1.00	0.00	0.00
2275	1.00	0.00	0.00	3282	1.00	0.00	0.00	3814	1.00	0.00	0.00
2279	1.00	0.00	0.00	3288	1.00	0.00	0.00	3815	1.00	0.00	0.00
2286	1.00	0.00	0.00	3291	1.00	0.00	0.00	3818	1.00	0.00	0.00
2316	0.97	0.03	0.00	3297	1.00	0.00	0.00	3819	1.00	0.00	0.00
2317	1.00	0.00	0.00	3298	1.00	0.00	0.00	3823	1.00	0.00	0.00
2338	1.00	0.00	0.00	3300	1.00	0.00	0.00	3833	1.00	0.00	0.00
2447	1.00	0.00	0.00	3302	1.00	0.00	0.00	3838	1.00	0.00	0.00
2467	1.00	0.00	0.00	3303	1.00	0.00	0.00	3840	0.00	1.00	0.00
2481	1.00	0.00	0.00	3318	1.00	0.00	0.00	3870	1.00	0.00	0.00
2546	1.00	0.00	0.00	3329	1.00	0.00	0.00	3871	1.00	0.00	0.00
2547	1.00	0.00	0.00	3349	1.00	0.00	0.00	3880	1.00	0.00	0.00
2549	1.00	0.00	0.00	3357	1.00	0.00	0.00	3884	1.00	0.00	0.00

(*continued on next page*)

Table B2
(*continued*)

ID	M1	M2	M3	ID	M1	M2	M3	ID	M1	M2	M3
2551	1.00	0.00	0.00	3379	1.00	0.00	0.00	3887	1.00	0.00	0.00
2554	1.00	0.00	0.00	3380	1.00	0.00	0.00	3889	1.00	0.00	0.00
2566	1.00	0.00	0.00	3382	1.00	0.00	0.00	3891	1.00	0.00	0.00
2573	1.00	0.00	0.00	3388	1.00	0.00	0.00	3892	1.00	0.00	0.00
2576	1.00	0.00	0.00	3399	1.00	0.00	0.00	3894	1.00	0.00	0.00
2589	1.00	0.00	0.00	3400	1.00	0.00	0.00	3895	1.00	0.00	0.00
2596	1.00	0.00	0.00	3401	1.00	0.00	0.00	3897	1.00	0.00	0.00
2611	1.00	0.00	0.00	3407	1.00	0.00	0.00	3898	1.00	0.00	0.00
2633	1.00	0.00	0.00	3410	1.00	0.00	0.00	3899	1.00	0.00	0.00
2676	1.00	0.00	0.00	3418	1.00	0.00	0.00	3903	1.00	0.00	0.00
2692	1.00	0.00	0.00	3424	1.00	0.00	0.00	3917	1.00	0.00	0.00
2715	1.00	0.00	0.00	3425	1.00	0.00	0.00	3928	1.00	0.00	0.00
2719	1.00	0.00	0.00	3438	1.00	0.00	0.00	3932	1.00	0.00	0.00
2727	1.00	0.00	0.00	3441	1.00	0.00	0.00	3939	1.00	0.00	0.00
2730	1.00	0.00	0.00	3454	1.00	0.00	0.00	3940	1.00	0.00	0.00
2759	1.00	0.00	0.00	3456	1.00	0.00	0.00	3948	1.00	0.00	0.00
2766	1.00	0.00	0.00	3462	1.00	0.00	0.00	3952	1.00	0.00	0.00
2769	0.02	0.00	0.98	3463	1.00	0.00	0.00	3959	1.00	0.00	0.00
2771	1.00	0.00	0.00	3464	1.00	0.00	0.00	3960	1.00	0.00	0.00
2772	1.00	0.00	0.00	3465	1.00	0.00	0.00	3971	1.00	0.00	0.00
2779	1.00	0.00	0.00	3467	1.00	0.00	0.00	4008	1.00	0.00	0.00
2794	1.00	0.00	0.00	3492	1.00	0.00	0.00	4011	1.00	0.00	0.00
2803	1.00	0.00	0.00	3493	1.00	0.00	0.00	4017	1.00	0.00	0.00
2804	1.00	0.00	0.00	3514	1.00	0.00	0.00	4027	1.00	0.00	0.00
2812	1.00	0.00	0.00	3522	1.00	0.00	0.00	4037	1.00	0.00	0.00
2864	1.00	0.00	0.00	3523	1.00	0.00	0.00	4038	1.00	0.00	0.00
2870	1.00	0.00	0.00	3526	1.00	0.00	0.00	4058	1.00	0.00	0.00
2874	1.00	0.00	0.00	3529	1.00	0.00	0.00	4082	1.00	0.00	0.00
2876	1.00	0.00	0.00	3530	1.00	0.00	0.00	4115	1.00	0.00	0.00
2877	0.00	1.00	0.00	3531	1.00	0.00	0.00	4118	1.00	0.00	0.00
2887	1.00	0.00	0.00	3544	1.00	0.00	0.00	4119	1.00	0.00	0.00
2892	1.00	0.00	0.00	3552	0.99	0.01	0.00	4128	1.00	0.00	0.00
2897	1.00	0.00	0.00	3562	1.00	0.00	0.00	4132	1.00	0.00	0.00
2898	1.00	0.00	0.00	3565	1.00	0.00	0.00	4136	1.00	0.00	0.00
2899	1.00	0.00	0.00	3566	1.00	0.00	0.00	4138	1.00	0.00	0.00
2901	1.00	0.00	0.00	3568	1.00	0.00	0.00	4164	1.00	0.00	0.00
2915	1.00	0.00	0.00	3573	1.00	0.00	0.00	4186	1.00	0.00	0.00
2917	1.00	0.00	0.00	3584	1.00	0.00	0.00	4190	1.00	0.00	0.00
2919	1.00	0.00	0.00	3585	1.00	0.00	0.00	4194	1.00	0.00	0.00
2934	1.00	0.00	0.00	3589	1.00	0.00	0.00	5000	1.00	0.00	0.00
2935	1.00	0.00	0.00	3602	1.00	0.00	0.00	5788	1.00	0.00	0.00
2936	1.00	0.00	0.00	3611	1.00	0.00	0.00	5828	1.00	0.00	0.00
2937	0.00	1.00	0.00	3613	1.00	0.00	0.00	5829	1.00	0.00	0.00
2938	1.00	0.00	0.00	3645	1.00	0.00	0.00	5841	1.00	0.00	0.00
2939	1.00	0.00	0.00	3659	1.00	0.00	0.00	5986	1.00	0.00	0.00
2942	1.00	0.00	0.00	3662	1.00	0.00	0.00	5995	1.00	0.00	0.00
2949	1.00	0.00	0.00	3665	1.00	0.00	0.00	6746	1.00	0.00	0.00
2962	1.00	0.00	0.00	3671	1.00	0.00	0.00	3711	1.00	0.00	0.00
2973	1.00	0.00	0.00	3680	1.00	0.00	0.00	3712	1.00	0.00	0.00
2985	1.00	0.00	0.00	3699	1.00	0.00	0.00	3714	1.00	0.00	0.00
3011	1.00	0.00	0.00	3709	1.00	0.00	0.00	3719	1.00	0.00	0.00
3042	1.00	0.00	0.00	3710	1.00	0.00	0.00	3721	0.18	0.82	0.00
3054	0.10	0.90	0.00	3724	1.00	0.00	0.00	3723	1.00	0.00	0.00
3065	1.00	0.00	0.00	3738	1.00	0.00	0.00				
Marg. Dens.	−2828.2	−2839.4	−2845.8	Marg dens.	−2828.2	−2839.4	−2845.8	Marg. Dens.	−2828.2	−2839.4	−2845.8

Table B3
Subject-specific predictions for the survival and longitudinal outcomes at the final visit time and next three time points for the three joint models with non-zero posterior weights for model 2 and model 3.

Id	Time	Model 1 long Pred	Model 2 Long Pred	Model 3 Long Pred	BMA Long pred	Surv time	Model 1 Surv Pred	Model 2 Surv Pred	Model 3 Surv Pred	BMA Survival predicti
996	18.00	24.03	24.04	24.07	24.04	18.00	1.00	1.00	1.00	1.00
	19.29	24.11	24.13	24.15	24.13	19.02	0.99	0.99	0.99	0.99
	20.57	24.17	24.21	24.22	24.21	20.66	0.99	0.98	0.99	0.98
	21.86	24.21	24.27	24.27	24.27	22.30	0.98	0.98	0.98	0.98
2161	36.00	19.24	19.00	18.64	19	36.00	1.00	1.00	1.00	1.00
	36.54	19.11	18.87	18.47	18.87	37.05	0.99	0.99	0.98	0.99
	37.07	18.98	18.73	18.30	18.73	38.69	0.97	0.97	0.96	0.97
	37.61	18.84	18.59	18.12	18.59	40.32	0.95	0.96	0.94	0.95
2204	24.00	25.29	25.27	25.29	25.27	24.00	1.00	1.00	1.00	1.00
	25.04	25.28	25.26	25.27	25.26	25.58	0.98	0.98	0.98	0.98
	26.07	25.24	25.23	25.22	25.23	27.22	0.96	0.97	0.96	0.96
	27.11	25.18	25.18	25.14	25.18	28.86	0.94	0.95	0.94	0.94
2248	24.00	17.20	17.34	17.32	17.20	24.00	1.00	1.00	1.00	1.00
	25.03	17.21	17.36	17.34	17.21	25.57	0.98	0.98	0.97	0.98
	26.07	17.21	17.37	17.35	17.21	27.22	0.95	0.96	0.94	0.96
	27.11	17.20	17.38	17.36	17.20	28.86	0.93	0.94	0.91	0.94
2769	24.00	29.23	29.40	29.34	29.23	24.00	1.00	1.00	1.00	1.00
	25.03	29.16	29.36	29.29	29.16	25.57	0.99	0.99	0.98	0.99
	26.07	29.07	29,30	29.21	29.07	27.21	0.97	0.98	0.97	0.97
	27.11	28.97	29.22	29.13	28.97	28.85	0.96	0.96	0.95	0.96
2877	30.00	25.74	25.61	25.46	25.74	30.00	1.00	1.00	1.00	1.00
	30.78	25.63	25.51	25.31	25.63	30.49	0.99	0.99	0.99	0.99
	31.57	25.51	25.39	25.15	25.51	32.13	0.94	0.95	0.95	0.94
	32.36	25.37	25.27	24.97	25.37	33.77	0.90	0.91	0.91	0.90
2937	24.00	22.87	22.79	22.77	22.87	24.00	1.00	1.00	1.00	1.00
	25.03	22.89	22.81	22.77	22.89	25.57	0.98	0.99	0.98	0.99
	26.07	22.89	22.81	22.74	22.89	27.21	0.97	0.97	0.97	0.97
	27.11	22.86	22.79	22.70	22.86	28.85	0.96	0.96	0.95	0.96
3137	18.00	28.33	28.07	28.29	28.33	18.00	1.00	1.00	1.00	1.00
	19.28	28.61	28.34	28.53	28.61	19.02	0.99	0.99	0.99	0.99
	20.57	28.82	28.54	28.71	28.82	20.66	0.98	0.98	0.99	0.98
	21.86	28.99	28.69	28.83	28.99	22.30	0.96	0.96	0.98	0.97
3236	24.00	21.29	21.34	21.28	21.29	24.00	1.00	1.00	1.00	1.00
	25.04	21.33	21.38	21.31	21.33	25.58	0.99	0.99	0.99	0.99
	26.07	21.35	21.42	21.34	21.35	27.21	0.98	0.98	0.98	0.98
	27.11	21.37	21.44	21.36	21.37	28.85	0.98	0.98	0.97	0.98
3758	0.00	18.99	18.93	18.99	18.94	0.00	1.00	1.00	1.00	1.00
	2.03	19.84	19.87	19.82	19.86	1.00	0.96	0.97	0.95	0.96
	4.07	20.66	20.78	20.62	20.76	2.64	0.89	0.91	0.89	0.91
	6.11	21.41	21.60	21.34	21.57	4.27	0.84	0.87	0.84	0.86
3790	42.00	18.63	18.83	19.12	18.63	42.00	1.00	1.00	1.00	1.00
	42.28	18.63	18.84	19.14	18.63	43.60	0.99	0.99	0.99	0.99
	42.57	18.64	18.85	19.16	18.64	45.24	0.99	0.98	0.98	0.99
	42.86	18.65	18.86	19.18	18.65	46.88	0.98	0.98	0.98	0.98
3840	6.00	16.24	16.25	16.24	16.24	6.00	1.00	1.00	1.00	1.00
	7.78	16.93	16.94	16.93	16.93	7.55	0.95	0.95	0.94	0.95
	9.57	17.54	17.56	17.54	17.54	9.19	0.89	0.91	0.89	0.89
	11.36	18.06	18.09	18.07	18.06	10.83	0.85	0.87	0.85	0.85

Appendix C: Plots

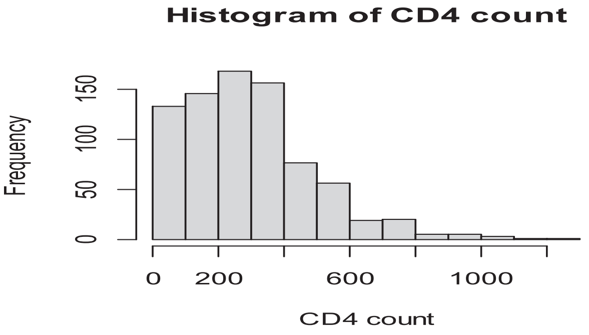

Fig. C1. Histogram of observed CD4 count data.

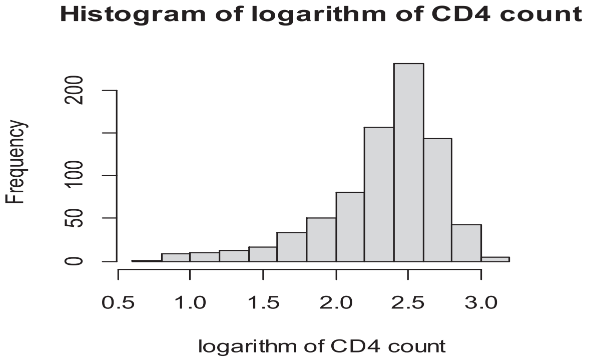

Fig. C2. Histogram of logarithm of observed CD4 count data.

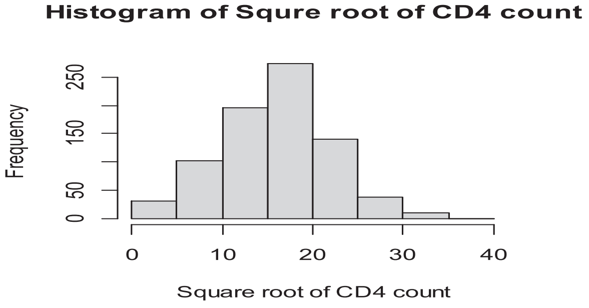

Fig. C3. Histogram of square root of observed CD4 count data.

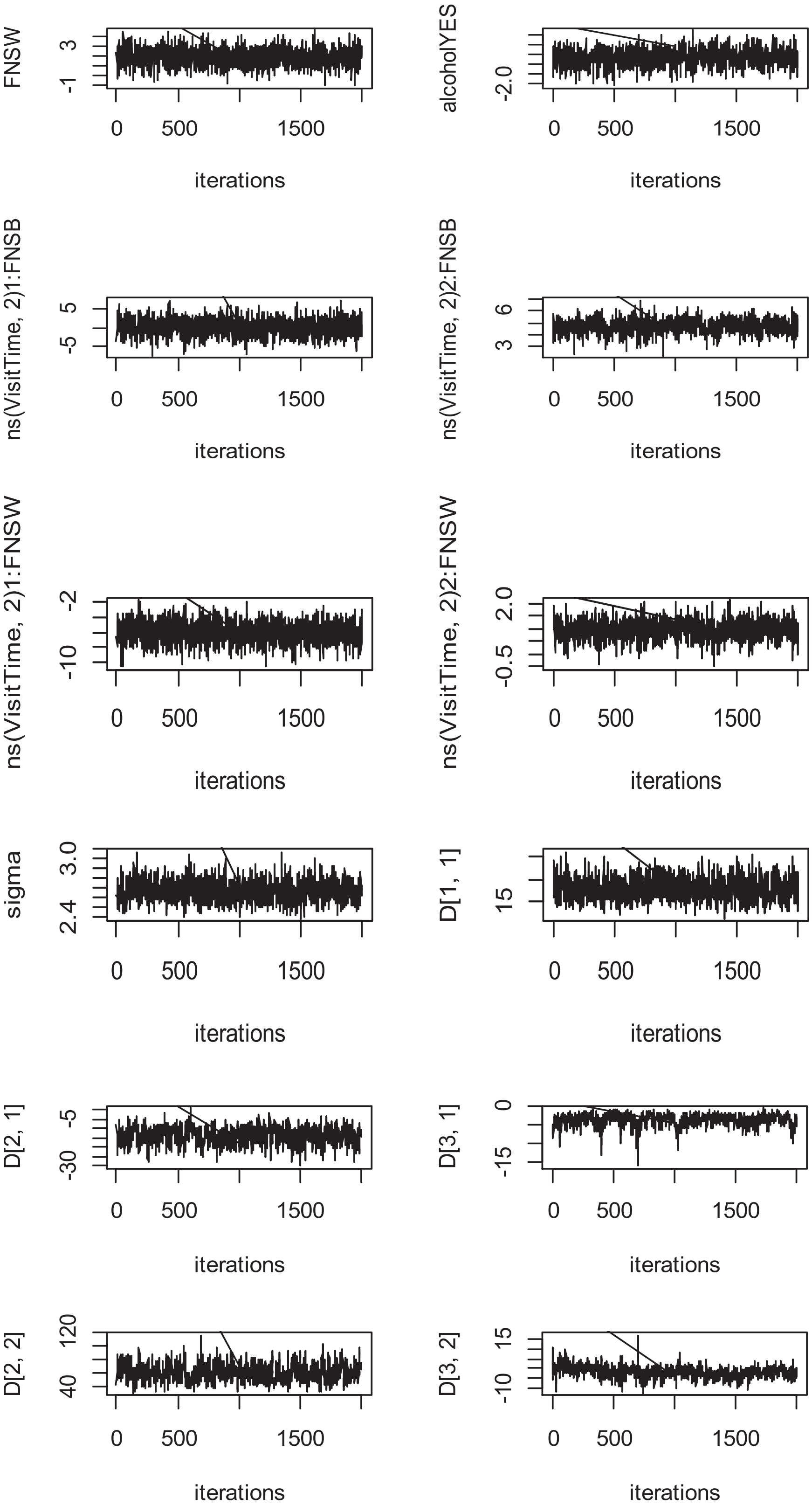

Fig. C4. MCMC diagnostic plots.

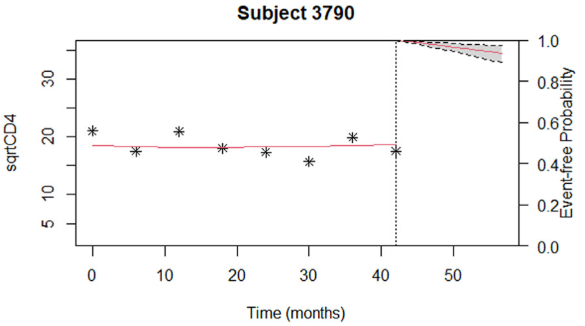

Fig. C5. Dynamic survival probabilities for patient 3790 from HIV data set during follow up.

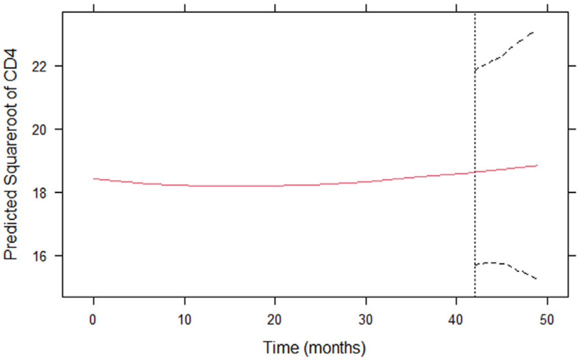

Fig. C6. Dynamic predictions of longitudinal responses for patient 3790 from HIV data set.

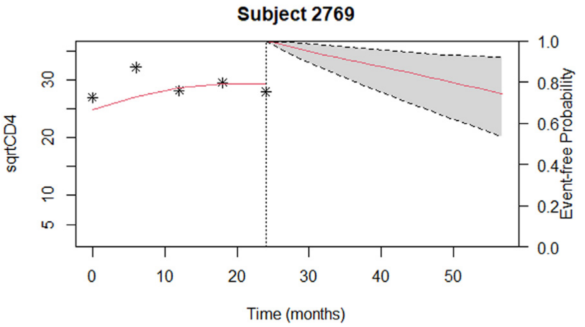

Fig. C7. Dynamic survival probabilities for patient 2769 from HIV data set during follow up.

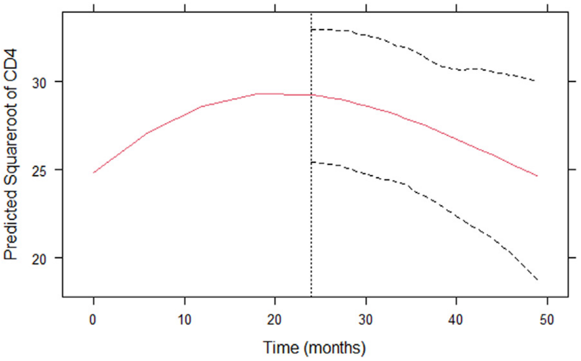

Fig. C8. Dynamic predictions of longitudinal responses for patient 2769 from HIV data set.
